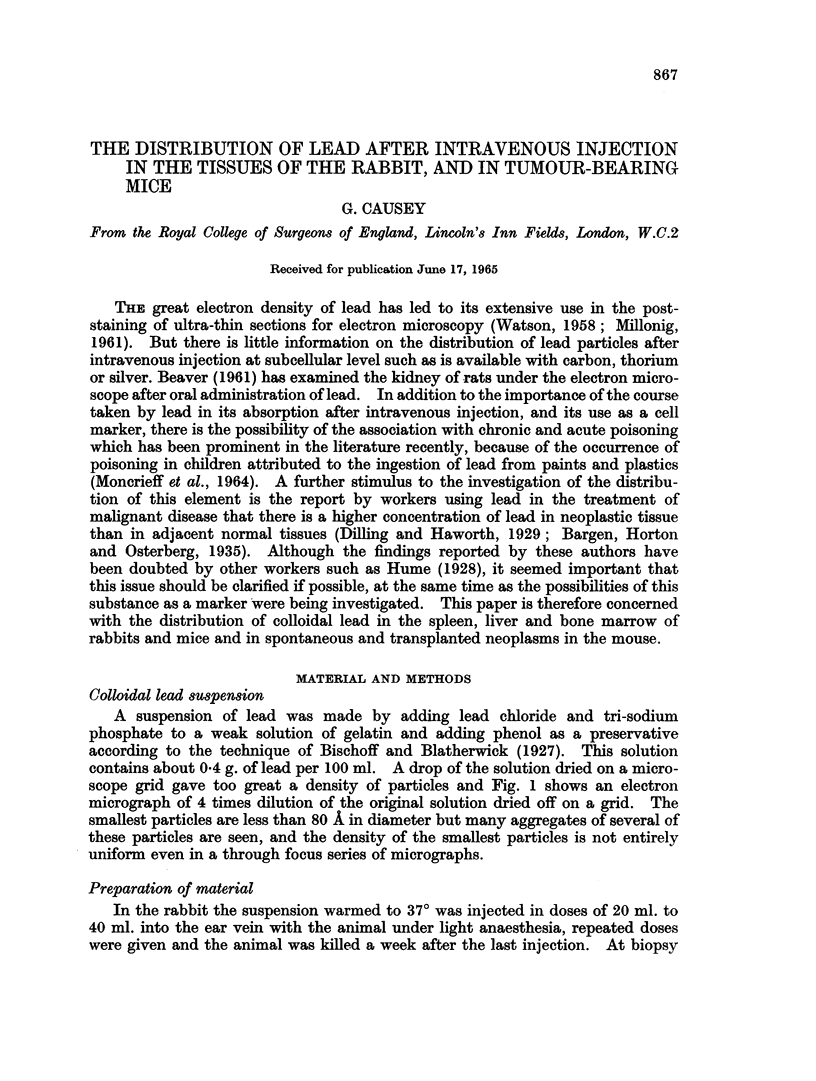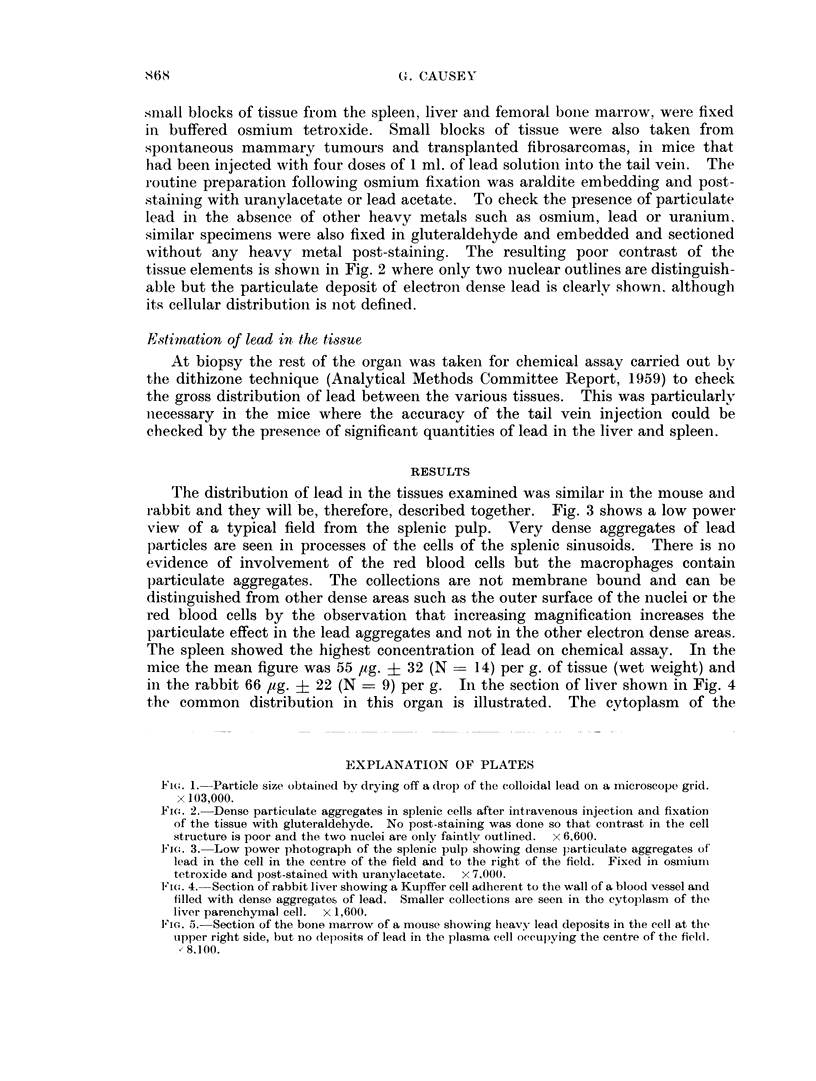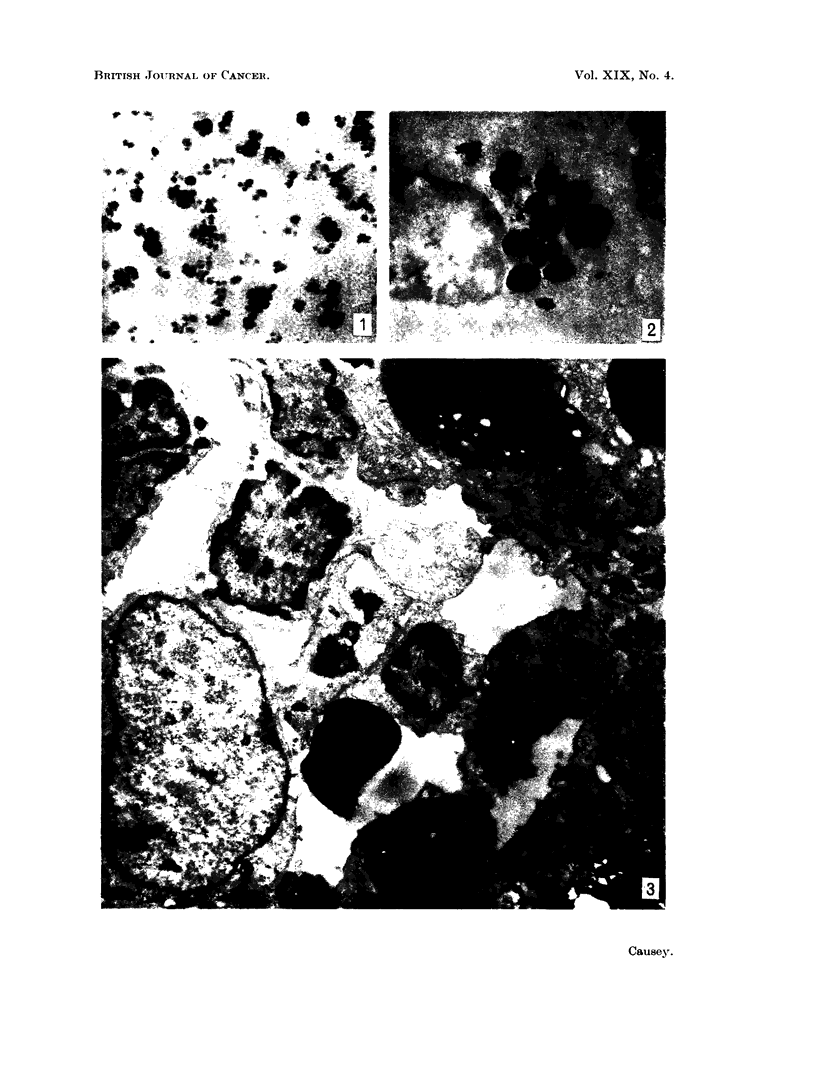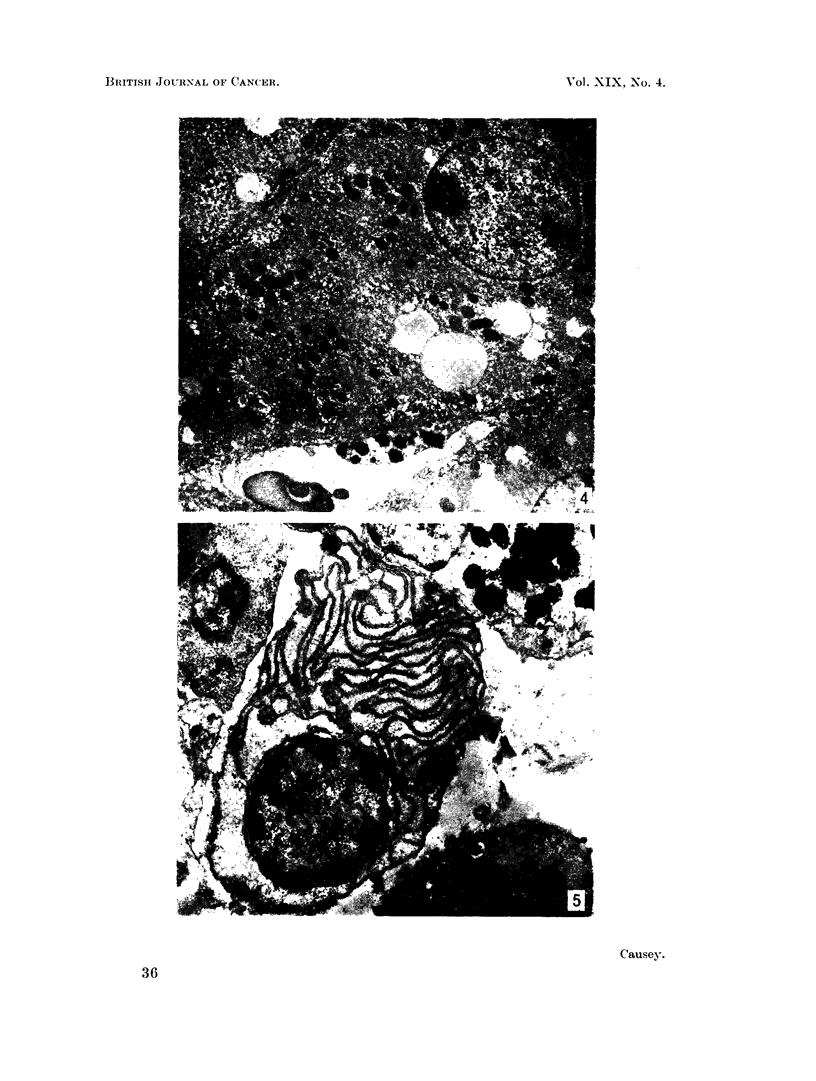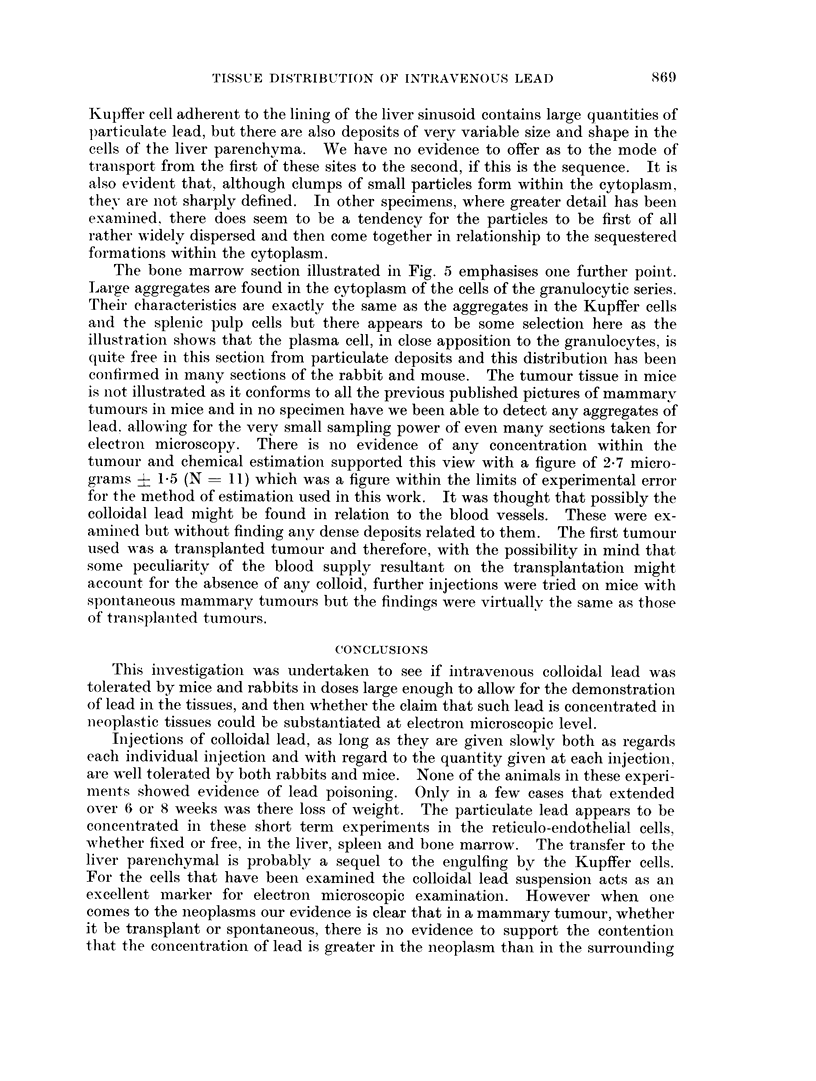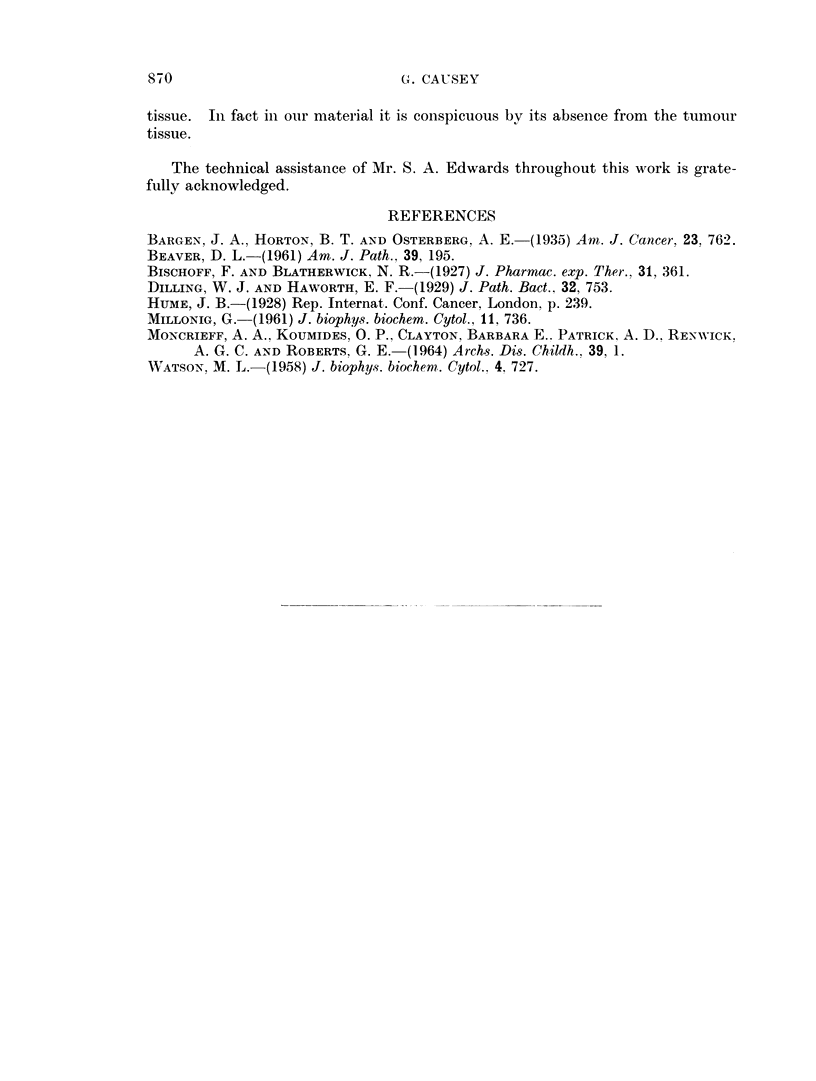# The distribution of lead after intravenous injection in the tissues of the rabbit, and in tumour-bearing mice.

**DOI:** 10.1038/bjc.1965.100

**Published:** 1965-12

**Authors:** G. Causey

## Abstract

**Images:**


### THE DISTRIBUTION OF LEAD AFTER INTRAVENOUS INJECTION IN THE TISSUES OF THE RABBIT, AND IN TUMOUR-BEARING MICE

G. CAUSEY

*From the Royal College of Surgeons of England, Lincoln's Inn Fields, London, W.C.2*

Received for publication June 17, 1965

THE great electron density of lead has led to its extensive use in the post-staining of ultra-thin sections for electron microscopy (Watson, 1958 ; Millonig, 1961). But there is little information on the distribution of lead particles after intravenous injection at subcellular level such as is available with carbon, thorium or silver. Beaver (1961) has examined the kidney of rats under the electron microscope after oral administration of lead. In addition to the importance of the course taken by lead in its absorption after intravenous injection, and its use as a cell marker, there is the possibility of the association with chronic and acute poisoning which has been prominent in the literature recently, because of the occurrence of poisoning in children attributed to the ingestion of lead from paints and plastics (Moncrieff *et al.*, 1964). A further stimulus to the investigation of the distribution of this element is the report by workers using lead in the treatment of malignant disease that there is a higher concentration of lead in neoplastic tissue than in adjacent normal tissues (Dilling and Haworth, 1929 ; Bargen, Horton and Osterberg, 1935). Although the findings reported by these authors have been doubted by other workers such as Hume (1928), it seemed important that this issue should be clarified if possible, at the same time as the possibilities of this substance as a marker were being investigated. This paper is therefore concerned with the distribution of colloidal lead in the spleen, liver and bone marrow of rabbits and mice and in spontaneous and transplanted neoplasms in the mouse.

### MATERIAL AND METHODS

### *Colloidal lead suspension*

A suspension of lead was made by adding lead chloride and tri-sodium phosphate to a weak solution of gelatin and adding phenol as a preservative according to the technique of Bischoff and Blatherwick (1927). This solution contains about 0·4 g. of lead per 100 ml. A drop of the solution dried on a microscope grid gave too great a density of particles and Fig. 1 shows an electron micrograph of 4 times dilution of the original solution dried off on a grid. The smallest particles are less than 80 Å in diameter but many aggregates of several of these particles are seen, and the density of the smallest particles is not entirely uniform even in a through focus series of micrographs.

### *Preparation of material*

In the rabbit the suspension warmed to 37° was injected in doses of 20 ml. to 40 ml. into the ear vein with the animal under light anaesthesia, repeated doses were given and the animal was killed a week after the last injection. At biopsy small blocks of tissue from the spleen, liver and femoral bone marrow, were fixed in buffered osmium tetroxide. Small blocks of tissue were also taken from spontaneous mammary tumours and transplanted fibrosarcomas, in mice that had been injected with four doses of 1 ml. of lead solution into the tail vein. The routine preparation following osmium fixation was araldite embedding and post-staining with uranylacetate or lead acetate. To check the presence of particulate lead in the absence of other heavy metals such as osmium, lead or uranium, similar specimens were also fixed in gluteraldehyde and embedded and sectioned without any heavy metal post-staining. The resulting poor contrast of the tissue elements is shown in Fig. 2 where only two nuclear outlines are distinguishable but the particulate deposit of electron dense lead is clearly shown, although its cellular distribution is not defined.

*Estimation of lead in the tissue*

At biopsy the rest of the organ was taken for chemical assay carried out by the dithizone technique (Analytical Methods Committee Report, 1959) to check the gross distribution of lead between the various tissues. This was particularly necessary in the mice where the accuracy of the tail vein injection could be checked by the presence of significant quantities of lead in the liver and spleen.

### RESULTS

The distribution of lead in the tissues examined was similar in the mouse and rabbit and they will be, therefore, described together. Fig. 3 shows a low power view of a typical field from the splenic pulp. Very dense aggregates of lead particles are seen in processes of the cells of the splenic sinusoids. There is no evidence of involvement of the red blood cells but the macrophages contain particulate aggregates. The collections are not membrane bound and can be distinguished from other dense areas such as the outer surface of the nuclei or the red blood cells by the observation that increasing magnification increases the particulate effect in the lead aggregates and not in the other electron dense areas. The spleen showed the highest concentration of lead on chemical assay. In the mice the mean figure was 55 $\mu$g. $\pm$ 32 (N = 14) per g. of tissue (wet weight) and in the rabbit 66 $\mu$g. $\pm$ 22 (N = 9) per g. In the section of liver shown in Fig. 4 the common distribution in this organ is illustrated. The cytoplasm of the

### EXPLANATION OF PLATES

FIG. 1.—Particle size obtained by drying off a drop of the colloidal lead on a microscope grid. $\times$ 103,000.

FIG. 2.—Dense particulate aggregates in splenic cells after intravenous injection and fixation of the tissue with gluteraldehyde. No post-staining was done so that contrast in the cell structure is poor and the two nuclei are only faintly outlined. $\times$ 6,600.

FIG. 3.—Low power photograph of the splenic pulp showing dense particulate aggregates of lead in the cell in the centre of the field and to the right of the field. Fixed in osmium tetroxide and post-stained with uranylacetate. $\times$ 7,000.

FIG. 4.—Section of rabbit liver showing a Kupffer cell adherent to the wall of a blood vessel and filled with dense aggregates of lead. Smaller collections are seen in the cytoplasm of the liver parenchymal cell. $\times$ 1,600.

FIG. 5.—Section of the bone marrow of a mouse showing heavy lead deposits in the cell at the upper right side, but no deposits of lead in the plasma cell occupying the centre of the field. $\times$ 8,100.

Causey.

Causey.

Kupffer cell adherent to the lining of the liver sinusoid contains large quantities of particulate lead, but there are also deposits of very variable size and shape in the cells of the liver parenchyma. We have no evidence to offer as to the mode of transport from the first of these sites to the second, if this is the sequence. It is also evident that, although clumps of small particles form within the cytoplasm, they are not sharply defined. In other specimens, where greater detail has been examined, there does seem to be a tendency for the particles to be first of all rather widely dispersed and then come together in relationship to the sequestered formations within the cytoplasm.

The bone marrow section illustrated in Fig. 5 emphasises one further point. Large aggregates are found in the cytoplasm of the cells of the granulocytic series. Their characteristics are exactly the same as the aggregates in the Kupffer cells and the splenic pulp cells but there appears to be some selection here as the illustration shows that the plasma cell, in close apposition to the granulocytes, is quite free in this section from particulate deposits and this distribution has been confirmed in many sections of the rabbit and mouse. The tumour tissue in mice is not illustrated as it conforms to all the previous published pictures of mammary tumours in mice and in no specimen have we been able to detect any aggregates of lead, allowing for the very small sampling power of even many sections taken for electron microscopy. There is no evidence of any concentration within the tumour and chemical estimation supported this view with a figure of 2·7 micrograms $\pm$ 1·5 (N = 11) which was a figure within the limits of experimental error for the method of estimation used in this work. It was thought that possibly the colloidal lead might be found in relation to the blood vessels. These were examined but without finding any dense deposits related to them. The first tumour used was a transplanted tumour and therefore, with the possibility in mind that some peculiarity of the blood supply resultant on the transplantation might account for the absence of any colloid, further injections were tried on mice with spontaneous mammary tumours but the findings were virtually the same as those of transplanted tumours.

### CONCLUSIONS

This investigation was undertaken to see if intravenous colloidal lead was tolerated by mice and rabbits in doses large enough to allow for the demonstration of lead in the tissues, and then whether the claim that such lead is concentrated in neoplastic tissues could be substantiated at electron microscopic level.

Injections of colloidal lead, as long as they are given slowly both as regards each individual injection and with regard to the quantity given at each injection, are well tolerated by both rabbits and mice. None of the animals in these experiments showed evidence of lead poisoning. Only in a few cases that extended over 6 or 8 weeks was there loss of weight. The particulate lead appears to be concentrated in these short term experiments in the reticulo-endothelial cells, whether fixed or free, in the liver, spleen and bone marrow. The transfer to the liver parenchymal is probably a sequel to the engulfing by the Kupffer cells. For the cells that have been examined the colloidal lead suspension acts as an excellent marker for electron microscopic examination. However when one comes to the neoplasms our evidence is clear that in a mammary tumour, whether it be transplant or spontaneous, there is no evidence to support the contention that the concentration of lead is greater in the neoplasm than in the surrounding tissue. In fact in our material it is conspicuous by its absence from the tumour tissue.

The technical assistance of Mr. S. A. Edwards throughout this work is gratefully acknowledged.

### REFERENCES

BARGEN, J. A., HORTON, B. T. AND OSTERBERG, A. E.—(1935) *Am. J. Cancer*, **23**, 762.
BEAVER, D. L.—(1961) *Am. J. Path.*, **39**, 195.
BISCHOFF, F. AND BLATHERWICK, N. R.—(1927) *J. Pharmac. exp. Ther.*, **31**, 361.
DILLING, W. J. AND HAWORTH, E. F.—(1929) *J. Path. Bact.*, **32**, 753.
HUME, J. B.—(1928) Rep. Internat. Conf. Cancer, London, p. 239.
MILLONIG, G.—(1961) *J. biophys. biochem. Cytol.*, **11**, 736.
MONCRIEFF, A. A., KOUMIDES, O. P., CLAYTON, BARBARA E., PATRICK, A. D., RENWICK, A. G. C. AND ROBERTS, G. E.—(1964) *Archs. Dis. Childh.*, **39**, 1.
WATSON, M. L.—(1958) *J. biophys. biochem. Cytol.*, **4**, 727.